# Mycobacteria Exploit Host Hyaluronan for Efficient Extracellular Replication

**DOI:** 10.1371/journal.ppat.1000643

**Published:** 2009-10-30

**Authors:** Yukio Hirayama, Mamiko Yoshimura, Yuriko Ozeki, Isamu Sugawara, Tadashi Udagawa, Satoru Mizuno, Naoki Itano, Koji Kimata, Aki Tamaru, Hisashi Ogura, Kazuo Kobayashi, Sohkichi Matsumoto

**Affiliations:** 1 Department of Bacteriology, Osaka City University Graduate School of Medicine, Osaka, Osaka, Japan; 2 Sonoda Women's University, Amagasaki, Hyogo, Japan; 3 Mycobacterial Reference Center, The Research Institute of Tuberculosis, Kiyose, Tokyo, Japan; 4 Department of Molecular Oncology, Division of Molecular and Cellular Biology, Institute on Aging and Adaptation, Shinshu University Graduate School of Medicine, Nagano, Japan; 5 Research Complex for the Medicine Frontiers, Aichi Medical University, Yazako, Nagakute, Aichi, Japan; 6 Department of Infectious Diseases, Bacteriology Division, Osaka Prefectural Institute of Public Health, Osaka, Japan; 7 Department of Virology, Osaka City University Graduate School of Medicine, Osaka, Osaka, Japan; 8 Department of Immunology, National Institute of Infectious Diseases, Shinjuku-ku, Tokyo, Japan; Johns Hopkins School of Medicine, United States of America

## Abstract

In spite of the importance of hyaluronan in host protection against infectious organisms in the alveolar spaces, its role in mycobacterial infection is unknown. In a previous study, we found that mycobacteria interact with hyaluronan on lung epithelial cells. Here, we have analyzed the role of hyaluronan after mycobacterial infection was established and found that pathogenic mycobacteria can grow by utilizing hyaluronan as a carbon source. Both mouse and human possess 3 kinds of hyaluronan synthases (HAS), designated HAS1, HAS2, and HAS3. Utilizing individual HAS-transfected cells, we show that HAS1 and HAS3 but not HAS2 support growth of mycobacteria. We found that the major hyaluronan synthase expressed in the lung is HAS1, and that its expression was increased after infection with *Mycobacterium tuberculosis*. Histochemical analysis demonstrated that hyaluronan profoundly accumulated in the granulomatous legion of the lungs in *M. tuberculosis*-infected mice and rhesus monkeys that died from tuberculosis. We detected hyaluronidase activity in the lysate of mycobacteria and showed that it was critical for hyaluronan-dependent extracellular growth. Finally, we showed that L-Ascorbic acid 6-hexadecanoate, a hyaluronidase inhibitor, suppressed growth of mycobacteria *in vivo*. Taken together, our data show that pathogenic mycobacteria exploit an intrinsic host-protective molecule, hyaluronan, to grow in the respiratory tract and demonstrate the potential usefulness of hyaluronidase inhibitors against mycobacterial diseases.

## Author Summary

*Mycobacterium tuberculosis* and *Mycobacterium bovis* are major bacterial pathogens that kill approximately 2 million people annually by causing tuberculosis. The *M. tuberculosis* complex has several strategies to parasitize the host. After infection is established, these pathogens are rarely eliminated from the host, and nowadays approximately a third of the world's human population is infected with the *Mycobacterium tuberculosis* complex. The elucidation of the parasitic mechanisms of the *M. tuberculosis* complex is important for the development of novel strategies against the disease. The major portal entry of *M. tuberculosis* complex is through the respiratory tract. On the surface of the airway, hyaluronan retains bactericidal enzymes so that they are “ready-to-use”, protecting tissues from invading pathogens. Furthermore, fragmented hyaluronan produced as a result of infection is used by the immune system as a sensor of infection. Thus, hyaluronan plays a pivotal role in host defenses in the respiratory tract. However, in this study, we observed that the *M. tuberculosis* complex utilizes hyaluronan as a carbon source for multiplication. We also found that the *M. tuberculosis* complex has hyaluronidase activity and showed that it is critical for hyaluronan-dependent growth of the *M. tuberculosis* complex. This study demonstrates a novel parasitic mechanism of the *M. tuberculosis* complex and suggests that mycobacterial hyaluronidase is a potential drug target.

## Introduction

Infectious diseases caused by mycobacteria are serious threats to human health. Tuberculosis is caused by infection with mycobacteria, most frequently with *Mycobacterium tuberculosis* but also with *Mycobacterium bovis*, *Mycobacterium africanum*, *Mycobacterium microti*, and *Mycobacterium canetii* and kills around 2 million people annually. Leprosy is caused by *Mycobacterium leprae* and the globally registered prevalence of leprosy was around 22,000 cases at the beginning of 2006.

The major portal of entry for mycobacterial pathogens is through the respiratory tract. The primary phase of the infection begins with inhalation of bacteria, which are then phagocytosed by alveolar macrophages in the periphery of the lungs. In addition, several lines of evidence indicate that mycobacteria interact with epithelial cells in the respiratory tract [1]–[4]. The recent reports show the significant role of type II pneumocytes in the pathology of tuberculosis [3],[5],[6]. The onset of mycobacterial diseases frequently occurs after a long latent phase. Mycobacteria are an intracellular bacterium, multiplying within host cells, but also grow extracellularly [7],[8].

Macrophages phagocytose mycobacteria through interaction with several cell surface receptors, including complement receptors, mannose receptors, surfactant protein A, scavenger receptors, and Fc receptors [9]. By contrast, mycobacteria attaches to or invades lung epithelial cells through interactions with glycosaminoglycans (GAG) [10]. *M. tuberculosis*, *M. bovis* bacillus Calmette-Guerin (BCG), and *M. leprae* produce two types of GAG interacting adhesins, heparin-binding hemagglutinin (HBHA) [10],[11] and mycobacterial DNA-binding protein 1 (MDP1, also called histone-like protein and laminin-binding protein in *M. leprae*) [1],[12]. HBHA is secreted to the extracellular milieu from mycobacteria [13], whereas MDP1 is tightly attached on the mycobacterial cell wall [14].

We previously demonstrated that hyaluronan is a major portal for infection of mycobacteria into A549 human lung epithelial cells by interacting with MDP1 [1]. Hyaluronan is a nonsulfated linear GAG composed of thousands of repeating units of GlcNAc- (beta-1, 4)-GlcUA- (beta-1, 3) and is synthesized by 3 isoforms of hyaluronan synthases (HAS), designated HAS1, HAS2, and HAS3 in both mice and humans [15]–[18]. In vertebrates, hyaluronan is a ubiquitous structural component of the extracellular matrix, and is abundant in the chondral and vitreous tissues. Recent findings demonstrated that hyaluronan has a pivotal role in diverse dynamic biological functions such as embryonic development [19], cell migration [20],[21], tumor transformation, [22],[23], wound healing [24], and inflammation [25]–[27].

On the mucosal surface of the airway, hyaluronan retains bactericidal enzymes so that they are “ready-to-use”, protecting mucosal tissues from invading pathogens [28]. Furthermore, in the alveolar tracts, released fragmented HA stimulates innate immune responses by activating Toll-like receptor 2 and 4 dependent pathways and initiating lung inflammation [25]. By contrast, during resolution of respiratory inflammation, immuno-stimulatory hyaluronan is taken up via the hyaluronan receptor CD44 on alveolar macrophages [26]. Thus hyaluronan plays a pivotal role in host defenses in the respiratory tract, but its role in mycobacterial infection had not been elucidated so far. In this study, we analyzed the role of hyaluronan after mycobacterial infection was established.

## Results

### Hyaluronan enhances the extracellular growth of mycobacteria after attachment to A549 cells

**Figure 1:**
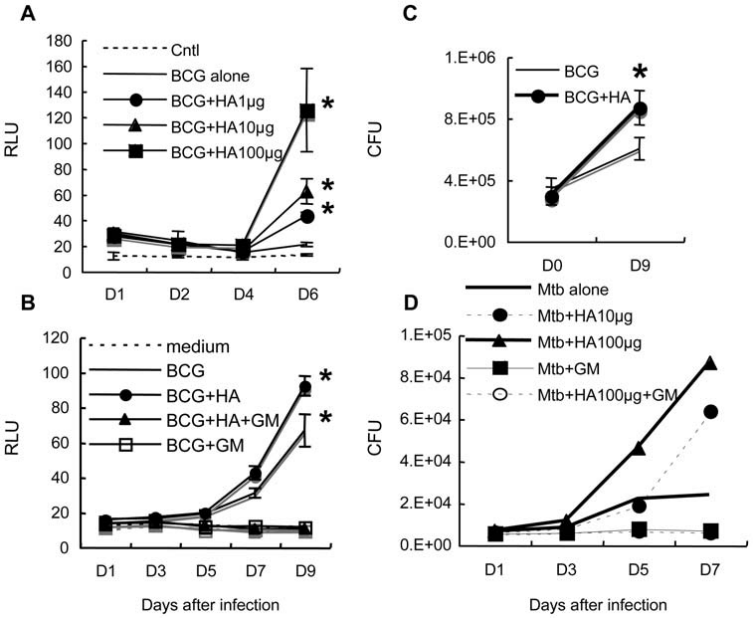
Effect of exogenously added hyaluronan on the growth of BCG and *M. tuberculosis* after infection of A549 cells. (A), A549 cells were infected with BCG-Luc for 16 hours at a multiplicity of infection (MOI) of 10. After removal of non-infected bacteria, different amounts of hyaluronan (HA) were added; 0 µg/200 µl (BCG alone), 1 µg/200 µl (BCG+HA1µg), 10 µg/200 µl (BCG+HA10µg), and 100 µg/200 µl (BCG+HA100µg) before culture at 37°C under 5% CO_2_. Cells were lysed by adding 5% Triton X (0.5% final) at each time point (1, 2, 4, and 6 days) and bacterial growth was monitored by luciferase activity. The results are expressed as mean±the standard deviation (*n* = 3). Relative luciferase unit (RLU). Cntl, control without BCG-Luc infection. For statistical analysis, a two-way ANOVA with Bonferroni Post tests were used to obtain *P*-values for each time point, comparing the various growth conditions to the control. *P<0.01. (B), Gentamicin (GM) treatment abrogated the growth of BCG-Luc after infection of A549 cells. A549 cells were infected with BCG-Luc for 16 hours at MOI of 10. After removal of non-infected bacteria, hyaluronan was added to be 500 µg/ml for some wells (BCG+HA, BCG+HA+GM) and cultured at 37°C under 5% CO_2_ in the presence or absence of 10 µg/ml GM (BCG+HA+GM, BCG+GM). Growth of BCG was monitored by luciferase activity. The results are expressed as mean±the standard deviation (*n* = 3). RLU. Cntl, control without BCG-Luc infection. (C), The enhancing effect of hyaluronan on BCG growth was confirmed by colony forming unit (CFU). A549 cells were infected with BCG-Luc for 16 hours at MOI of 10. After removal of non-infected bacteria, BCG-Luc was grown in the presence or absence of 50 µg/ml HA. Cells were lysed at each time point and serial 10-fold dilutions were plated in duplicate on Middlebrook 7H11 agar (Difco) supplemented with oleic acid, albumin, dextrose and catalase (Difco). After incubation for 3–4 weeks at 37°C, colonies were counted and the number of CFU was calculated per well (1 ml). The results are expressed as mean±the standard deviation (*n* = 6). (D), A549 cells were infected with *M. tuberculosis* H37Rv and then different amounts of hyaluronan (HA) were added; 0 µg/200 µl (Mtb alone), 10 µg/200 µl (Mtb+HA10µg), and 100 µg/200 µl (Mtb+HA100µg). Gentamycin (50 µg/ml) was added to some wells with (Mtb+HA100 µg+GM) or without (Mtb+GM) 100 µg/200 µl hyaluronan. Cells were lysed by adding 5% Triton X (0.5% final) and the number of viable bacteria was determined by plating dilutions of the samples for CFU on 7H11-OADC agar.

A549 cells, a type II human lung epithelial cell line, were exposed to recombinant BCG expressing luciferase (rBCG-Luc) under the control of the HSP60 promoter [14] at a multiplicity of infection (MOI) of 10 for 16 hours. Cells were then washed and various doses of hyaluronan added into the culture. Growth of BCG was monitored by luciferase activity at each time point, which is indicative of viable bacteria [14],[29]. We found that exogenously added hyaluronan enhances bacterial growth in a dose-dependent manner (Figure 1A). We also confirmed this effect by counting viable bacteria using a colony forming units (CFU) assay (Figure 1C).

In our experimental setting, around 60% of the bacteria adhere to the cell surface and the remaining 40% are internalized by the cells [1]. Therefore, we next examined whether hyaluronan enhances extracellular or intracellular growth by treatment with gentamicin, which kills extracellular but not intracellular bacteria. After infection, we added gentamicin (50 µg/ml) into the culture for 6 hours and then added hyaluronan after removing gentamicin. The results showed that gentamicin treatment abrogated the growth of BCG (Figure 1B), indicating that bacterial growth occurred extracellularly. The enhanced effect of hyaluronan on bacterial growth was also abolished by gentamicin treatment (Figure 1B). This suggests that hyaluronan enhances growth of BCG attached to these cells.

We next examined if the same effects of hyaluronan can be seen in *M. tuberculosis* growth after infection to A549 cells. We infected *M. tuberculosis* H37Rv to A549 cells, then added hyaluronan, and monitored growth by counting colony-forming units (CFU). Similar to the case of BCG, we found that presence of hyaluronan enhances the growth of *M. tuberculosis* in a dose dependent manner (Figure 1D). Gentamicin treatment also abrogated the growth of *M. tuberculosis* and growth-enhancing effect of hyaluronan.

### BCG utilizes hyaluronan as a carbon source

**Figure 2:**
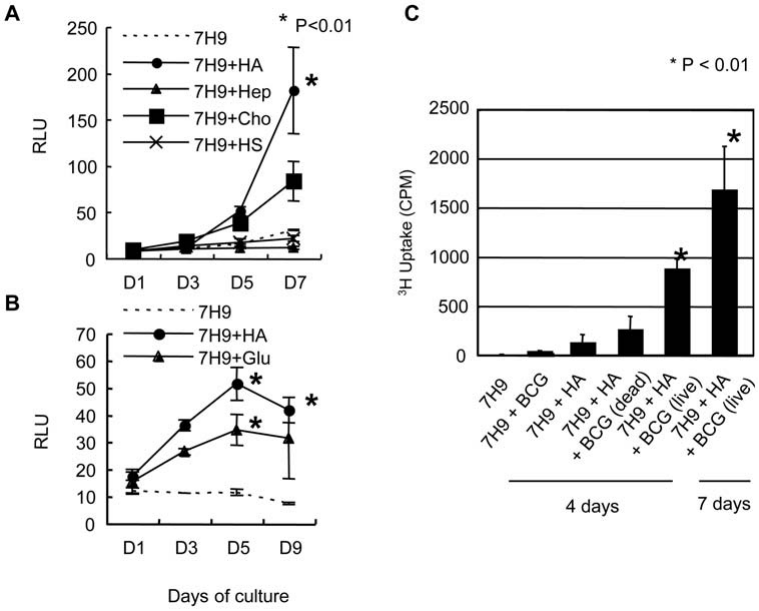
Effect of hyaluronan on BCG growth in carbon-starved 7H9 media. (A) (B), BCG-Luc was cultured in carbon-starved 7H9 media (7H9), or carbon-starved 7H9 media supplemented with 500 µg/ml of HA (7H9+HA), heparin (7H9+Hep), chondroitin sulfate C (7H9+Cho), heparan sulfate (7H9+HS), or glucose (7H9+Glu) at 37°C. Growth of BCG was monitored by luciferase activity. The results are expressed as mean±the standard deviation (*n* = 3). For statistical analysis, a two-way ANOVA with Bonferroni Post tests were used to obtain *P*-values for each time point, comparing the various growth conditions to the control. *P<0.01. (C), Uptake of ^3^H-hyaluronan (HA) by BCG in carbon-starved 7H9 media. Live and heat-killed BCG cells were cultured in carbon-starved 7H9 media in the presence or absence of ^3^H-labeled hyaluronan for 4 or 7 days. The uptake of ^3^H-laveled hyaluronan was measured by a gamma counter.

To determine why hyaluronan enhances the growth of BCG, we hypothesized that BCG can utilize it as a carbon source because hyaluronan is a polymer of disaccharides. We cultured BCG-Luc in 7H9 based carbon-starved broth in the presence (0.5 mg/ml) or absence of hyaluronan. As expected, in the carbon-starved media BCG did not grow, while the addition of hyaluronan supported the growth of BCG (Figure 2A), demonstrating that BCG can utilize hyaluronan as a carbon source.

We next compared hyaluronan with other GAG in terms of their growth supporting effect. BCG-Luc was cultured in 7H9-based carbon starved media or media including 0.5 mg/ml of each GAG as the sole carbon source. The results showed that BCG did not grow in the media supplemented with heparin or heparan sulfate. Both hyaluronan and chondroitin sulfate encouraged the growth, but hyaluronan sustained higher growth rates of BCG than chondroitin sulfate (Figure 2A). We also demonstrated that the growth supporting effect of hyaluronan is comparable to an equivalent amount of glucose (0.5 mg/ml) (Figure 2B).

In order to evaluate uptake of hyaluronan during hyaluronan-dependent growth of mycobacteria, we cultured BCG in the presence of ^3^H-labeled hyaluronan in the media containing hyaluronan as a sole carbon source. As shown in Figure 2C, live BCG incorporated hyaluronan, whereas heat-killed bacteria did not, showing actual uptake of hyaluronan into bacteria.

### *M. tuberculosis* can utilize hyaluronan as a carbon source, whereas neither *M. avium* nor *M. smegmatis* can

**Figure 3:**
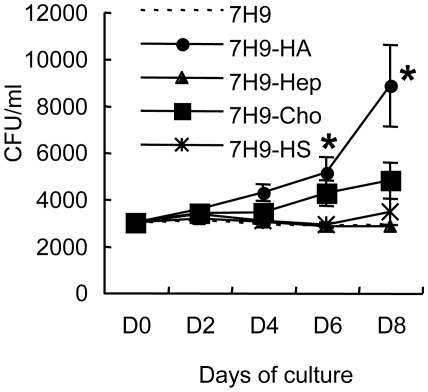
Effect of GAG on the growth of *M. tuberculosis* in carbon starved media. *M. tuberculosis* H37Rv was cultured in carbon-starved 7H9 media (7H9), or carbon-starved 7H9 media supplemented with 500 µg/ml of HA (7H9+HA), heparin (7H9+Hep), chondroitin sulfate C (7H9+Cho), or heparan sulfate (7H9+HS) at 37°C. Bacterial numbers were monitored by determining CFU at each time point. The results are expressed as mean±the standard deviation (*n* = 3). For statistical analysis, a two-way ANOVA with Bonferroni Post tests were used to obtain *P*-values for each time point, comparing the various growth conditions to the control. *P<0.01.

We next assessed the action of hyaluronan in the growth of virulent *M. tuberculosis* (strain H37Rv), and environmental mycobacterial species such as *M. smegmatis* (strain mc^2^155) and *M. avium* (ATCC25291). In carbon-starved media, none of the three strains grew. However, *M. tuberculosis* H37Rv, along with BCG, multiplied in the media containing hyaluronan as a sole carbon source while neither *M. smegmatis* nor *M. avium* proliferated. After 12 days culture, optimal density (OD) at 630 nm of *M. tuberculosis* culture increased to 0.32±0.038 from 0.01 (day 0). We then compared hyaluronan and other GAGs in terms of growth supportive effects on *M. tuberculosis*. Similar to the case of BCG, hyaluronan most effectively enhanced the growth of *M. tuberculosis* among tested GAGs (Figure 3).

### Detection of hyaluronidase activity in mycobacteria

**Figure 4:**
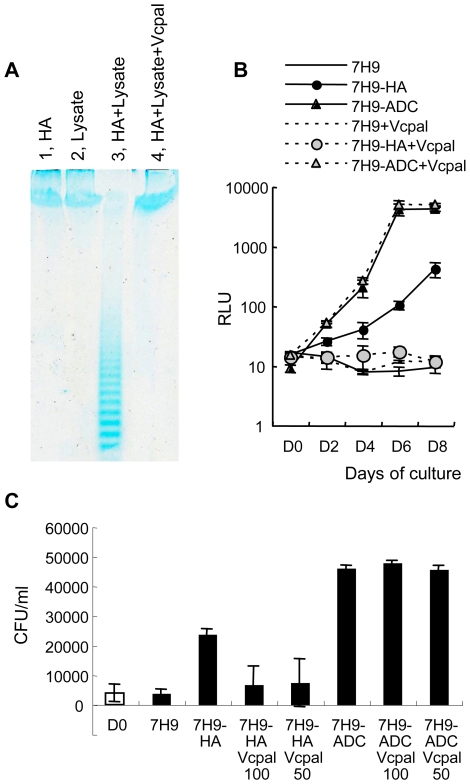
Hyaluronidase activity in mycobacteria and the effect of hyaluronidase inhibitor on hyaluronan-dependent growth of BCG and *M. tuberculosis*. (A), One mg/ml of hyaluronan and 700 µg/ml of BCG cell lysate was mixed and incubated for 3 days in the presence (HA+Lysate+Vcpal) or absence (HA+Lysate) of ascorbic palmitate (Vcpal), an inhibitor of hyaluronidase. As controls, hyaluronan alone (lane 1, HA) or BCG cell lysate alone (lane 2, Lysate) was treated in the same way. Hyaluronan was precipitated by ethanol after phenol extraction and resolved in water. Then hyaluronan was fractionated by PAGE gel electrophoresis and visualized by staining with alcian blue. (B), BCG-Luc (0.01 OD at 630 nm) was cultured in carbon-starved 7H9 media (7H9), media containing hyaluronan (500 µg/ml) as a sole carbon source (7H9-HA), or complete 7H9-ADC media (7H9-ADC) in the presence or absence of 25 µM Vcpal (+Vcpal), an inhibitor of hyaluronidase. The growth of bacteria was monitored by luciferase activity. RLU, relative luciferase unit (RLU). The results are expressed as mean±the standard deviation (*n* = 3). (C), The effect of Vcpal on the growth of *M. tuberculosis*. *M. tuberculosis* H37Rv was cultured in carbon starved 7H9 media (7H9), media containing 100 µg/ml hyaluronan as a sole carbon source (7H9-HA), or conventional 7H9-ADC media (7H9-ADC) with or without 50 (50) or 100 (100) µM of Vcpal for 8 days (closed bars). Bacterial number was determined by plating dilutions for CFU on 7H9-OADC agar and compared to that of Time 0 (D0, open bar).

Because hyaluronan is a long chain consisting of the repeat of two monosaccharides at over 2×10^5^ Da, we hypothesized that extracellular cleavage of the polymer would be required before taken up by cells. Therefore, we next assessed hyaluronidase activity in mycobacteria. Hyaluronan was incubated in the presence or absence of cell lysates derived from BCG before precipitation by phenol/chloroform extraction. Precipitates were then fractionated by polyacrylamide gel electrophoresis (PAGE) and visualized by alcian blue staining as described previously [30]. Hyaluronan was separated into discrete ladder-like bands by electrophoresis after incubation with BCG lysate (Figure 4A), demonstrating that BCG possesses hyaluronidase activity.

### Hyaluronidase activity is critical for hyaluronan-dependent growth

We then addressed whether hyaluronidase activity is crucial for hyaluronan -dependent growth of mycobacteria. L-Ascorbic acid 6-hexadecanoate (Vcpal) is shown to be a potent inhibitor of hyaluronidase [31]. We investigated the effect of Vcpal on hyaluronidase activity of BCG and found that hyaluronidase activity was abolished in the presence of 25 µM Vcpal (Figure 4A, lane 4).

We next examined the effects of Vcpal on the growth of BCG. BCG-Luc was cultured in modified 7H9 media containing hyaluronan (0.5 mg/L) as the sole carbon source or 7H9-ADC complete media, which contains Tween 80, glycerol, and dextrose as carbon sources and BSA. We found that 25 µM Vcpal did not change the growth rate of BCG in 7H9-ADC complete media, while it abolished the growth of BCG in the media containing hyaluronan as the sole carbon source (Figure 4B).

We also examined the effect of Vcpal on the growth of *M. tuberculosis*. *M. tuberculosis* H37Rv was cultured in the media with or without Vcpal (50 and 100 µM). Vcpal suppressed the growth of *M. tuberculosis* in the media containing hyaluronan as a sole carbon source but not the growth in conventional 7H9-ADC media (Figure 4C). Other hyaluronidase inhibitors, such as apigenin and quercetin [32], also inhibited hyaluronan dependent growth of *M. tuberculosis* as shown in Figure S1. These results indicate that hyaluronidase activity is essential for both BCG and *M. tuberculosis* when utilizing hyaluronan as a carbon source.

### Vcpal blocks growth of BCG after attachment to A549 cells

We next examined whether Vcpal suppresses the enhancing effect of hyaluronan on the growth of BCG after attachment to A549 epithelial cells. After exposure to BCG-Luc, hyaluronan was added with or without Vcpal (25 µM) into the culture and growth of BCG was monitored by measuring luciferase activity. After 6 days culture, RLU values of BCG-Luc increased to 36.6±7.5 RLU or 52.6±18.7 RLU in the absence or presence of hyaluronan, respectably. Adding Vcpal abrogated the enhanced effects of hyaluronan (29.3±2 RLU), demonstrating that BCG utilized exogenously added hyaluronan as a carbon source after infection to A549 cells.

### BCG and *M. tuberculosis* efficiently utilize hyaluronan synthesized by HAS1 and HAS3

This work so far on the growth of mycobacteria has been performed with hyaluronan purified from human umbilical cord (Sigma). In order to elucidate whether mycobacteria can use hyaluronan actually synthesized *in situ* by mammalian cells, we employed the previously established stable human HAS1–3 expressing rat 3Y1 fibroblasts [15]. 3Y1 rat fibroblasts do not produce detectable hyaluronan themselves but each transfectant produces different sized hyaluronan. Both HAS1 and HAS3 transfectants secret hyaluronan with broad size distributions with molecular masses between 2×10^5^ to ∼2×10^6^ Da, while the HAS2 transfectant secretes extremely large hyaluronan at an average molecular mass of >2×10^6^ Da [15]. We analyzed the level of hyaluronan production by utilizing a hyaluronan-binding protein (HABP)-based ELISA assay and confirmed that the HAS2 transfectant produced high levels of hyaluronan (235.7 µg/mL in the culture media), while the HAS3 transfectant synthesized the smallest amount of hyaluronan (15.9 µg/mL). The HAS1 transfectant produced moderate levels of hyaluronan (85.3 µg/mL), and the empty vector transfectant did not produce detectable amounts of hyaluronan.

**Figure 5:**
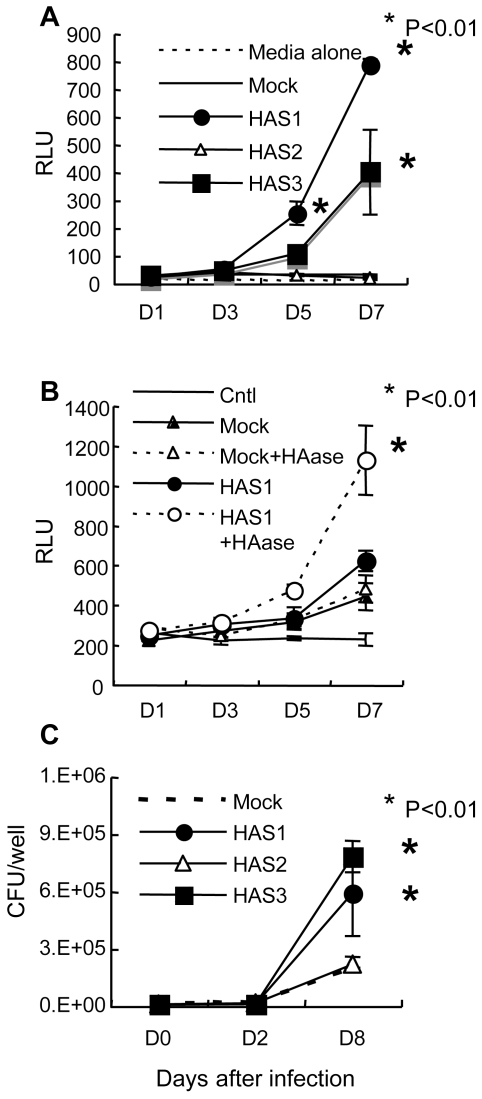
The effect of 3 hyaluronan synthases on the growth of BCG and *M. tuberculosis*. (A), Established transfectant cells (Rat 3Y1 fibroblasts) with control vector (Mock) or vector to express hyaluronan synthase 1 (HAS1), HAS2 (HAS2), or HAS3 (HAS3) were cultured in the presence of BCG-Luc or media alone. The growth of bacteria was monitored by luciferase activity. RLU, relative luciferase unit. The results are expressed as mean±the standard deviation (*n* = 3). For statistical analysis, a two-way ANOVA with Bonferroni Post tests were used to obtain *P*-values for each time point, comparing the various growth conditions to the control. *P<0.01. (B), Hyaluronidase (HAase) treatment enhances the growth of BCG after infection to HAS1-tranfected cells. After 16 hours exposure of BCG-Luc to transfected cells with control vector (Mock) or vector expressing HAS1 (HAS1), unbound bacteria were washed and cultured in the presence or absence of 2 units/ml of hyaluronidase (HAase). Bacterial growth was monitored by the luciferase activity (RLU). Cntl, HAS1-transfectant cells without infection of BCG-luc. The results are expressed as mean±the standard deviation (*n* = 3). For statistical analysis, a two-way ANOVA with Bonferroni Post tests were used to obtain *P*-values for each time point, comparing the various growth conditions to the control. *P<0.01. (C), The growth of *M. tuberculosis* H37Rv after infection to transfectant 3Y1 fibroblasts with control vector (Mock) or vector to express hyaluronan synthase 1 (HAS1), HAS2 (HAS2), or HAS3 (HAS3) was monitored by CFU. The results are expressed as mean±the standard deviation (*n* = 3). For statistical analysis, a two-way ANOVA with Bonferroni Post tests were used to obtain *P*-values for each time point, comparing the various growth conditions to the control. *P<0.01.

Each human HAS transfectant was exposed to BCG-Luc and the growth kinetics of the bacteria were monitored by luciferase activity. The results showed that BCG grew after attachment to 3Y1 cells transfected with HAS1 and HAS3 but not with HAS2 or empty vector (Figure 5A). In addition, we found that hyaluronidase treatment of HAS1 transfected cells enhanced the growth of BCG (Figure 5B). These results suggest that shorter sized chains of hyaluronan are preferential for BCG growth.

We also monitored the growth of *M. tuberculosis* H37Rv after infection to these HAS transfectant cells. Along with the case of BCG, HAS1 and HAS3 but not HAS2-tranfectants supported the growth of *M. tuberculosis* (Figure 5C).

### Production of hyaluronan in *M. tuberculosis*-infected lungs

**Figure 6:**
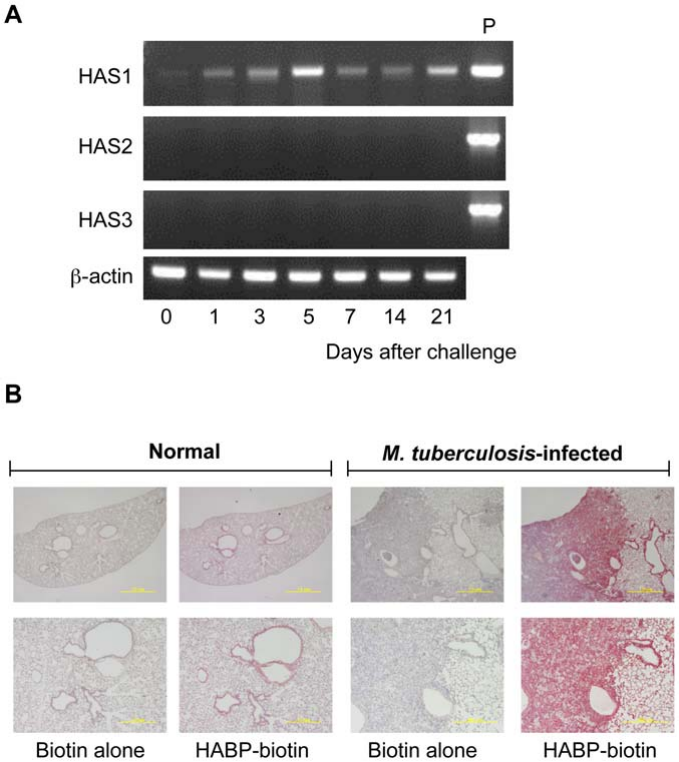
Production of hyaluronan during *M. tuberculosis* infection in mice. (A), BALB/c mice were aerogenically infected with *M. tuberculosis* H37Rv (around 10 CFU/lung). At the indicated periods, mice were euthanized and total RNA was extracted from the lungs. Transcription of each gene encoding HAS1, HAS2, HAS3 and beta-actin was analyzed by RT-PCR. Three mice were analyzed for each time point and representative data are presented. P, positive control of PCR employing the cDNA clone of each HAS gene as a template. (B), After euthanized, lungs from uninfected mice (Normal) or mice 21 days after infection with *M. tuberculosis* H37Rv (*M. tuberculosis* infected) were removed and histological sections were made by standard methods including formalin fixation, dehydration, and embedding in paraffin. Biotinylated hyaluronan-binding protein (HABP-biotin) was used to stain the hyaluronan in the lungs. Biotin alone was used as control straining (Biotin alone). Avidin-conjugated alkaline phosphatase and chromogen as the substrate were used to generate a red reaction product. Digital images of representative sites were acquired at ×20 (upper pictures) or ×100 (lower pictures) magnification. Experiments were performed at least three times using 5 mice for each group.

To see if hyaluronan is present at the site of infection of *M. tuberculosis*, we assessed the expression of hyaluronan synthases (HAS1, HAS2, and HAS3) in the lungs of BALB/c mice infected with the *M. tuberculosis* H37Rv strain, using the low-dose aerosol infection model. Total RNA was extracted from the lungs after 1, 3, 5, 7, 14, and 21 days of infection, and analyzed for HAS1, HAS2, and HAS3 mRNA transcription by reverse transcriptase-polymerase chain reaction (RT-PCR) (Figure 6A). The data showed that HAS1 mRNA expression increased after infection and was maintained at all time points (Figure 6A).

We next determined if hyaluronan is present in alveoli using biotin-conjugated hyaluronan-binding protein (HABP) and histochemical analysis. Before infection, hyaluronan was located on the surface of the airways and alveoli (Figure 6B). After *M. tuberculosis* infection, hyaluronan levels were profoundly increased and accumulated in the granulomatous legion (Figure 6B). Taken together, these data indicate that the major hyaluronan synthase in the lungs is HAS1 both before and after *M. tuberculosis* infection and hyaluronan accumulates in the tuberculosis lesion.

### Detection of hyaluronan in the lungs of rhesus monkeys that died of tuberculosis

**Figure 7:**
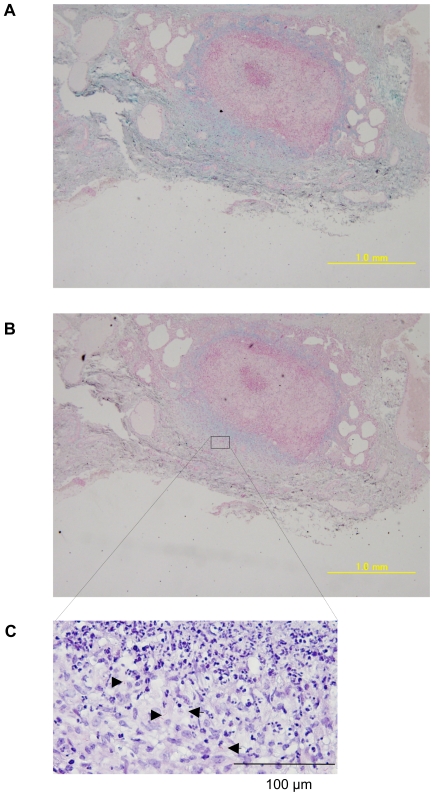
Presence of hyaluronan in the lungs of rhesus monkeys that died from tuberculosis. The lung sections were obtained from rhesus monkeys that had died of tuberculosis after challenge with 3,000 CFU/lung of *M. tuberculosis* H37Rv intratracheally. The sections were stained with alcian blue with (B) or without (A) pretreatment of hyaluronidase and counterstained with nuclear fast red. The section was also stained with Ziehl-Neelsen to demonstrate the presence of acid-fast bacilli (arrow heads) (C).

*M. tuberculosis*-infected mice had numerous sites of granulomatous inflammation in their lungs but in primates, tuberculosis granulomas are well-organized and tighter. We next studied hyaluronan in the lung granuloma of *M. tuberculosis* H37Rv-infected rhesus monkeys by staining with alcian blue, which is commonly used dye to detect GAG. The dye stained the surrounding region of well-organized granuloma (Figure 7A) and the staining was largely abolished by treatment with hyaluronidase (Figure 7B), showing that hyaluronan is a major GAG surrounding granuloma. Acid-fast bacilli (arrow heads in Figure 7C) were located in alcian blue stained areas, thus suggesting a strong correlation between the localization of the tubercle bacilli and hyaluronan.

### Vcpal suppresses mycobacterial growth *in vivo*

**Figure 8:**
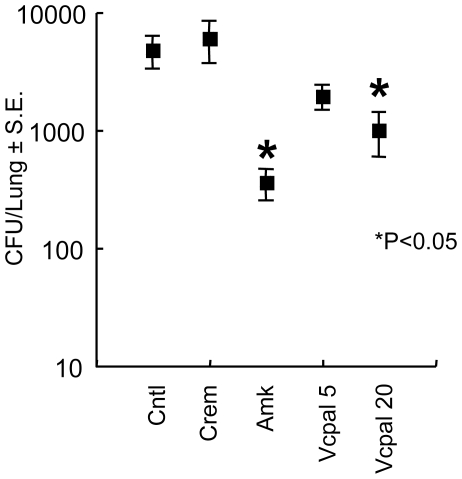
Vcpal suppresses the growth of mycobacteria in mouse lungs. BALB/c mice were infected with 10^6^ CFU of BCG (Pasteur) intravenously. One day after the challenge, mice were treated with amikacin (Amk) and Vcpal every day for 14 days. Two days after final treatment, mice were euthanized and their lungs were homogenized. Lung pastes were serially diluted and plated in duplicate on Middlebrook 7H11 OADC agars. After incubation for 3–4 weeks at 37°C, colonies were counted and the number of CFU was calculated per lung. For statistical analysis, a two-way ANOVA with Bonferroni Post tests were used to obtain *P*-values to determine the effect of Vcpal and amikacin on bacterial growth to the control. *P<0.05. Cntl, control mice without treatment.

Finally, we addressed the effect of Vcpal on the growth of BCG in BALB/c mice. Mice were infected with BCG intravenously through their tail veins. One day after BCG challenge, the hyaluronidase inhibitor Vcpal (0.4 or 1.64 mg/dose) was injected every day thorough the tail veins for 14 days. Two days after the final injection, the mice were euthanized and viable bacteria counts were determined by the CFU assay. As a positive control, we also treated mice with amikacin (Amk), which kills extracellular but not intracellular mycobacteria, by an intramuscular injection. The results showed that Vcpal apparently suppressed growth of BCG in the lungs, similar to Amk (Figure 8).

## Discussion

Although hyaluronan is crucial for both structural and physiological properties in the alveolar spaces, its role in mycobacterial infection was previously unknown. We demonstrated before that hyaluronan is the major attachment site of both BCG and *M. tuberculosis* in the infection of A549 cells, which itself produced hyaluronan [1] probably depending on HAS3 and HAS2 (Figure S2). In this study, we further extended our research and studied the role of hyaluronan after infection was established.

First, we examined the effect of hyaluronan on the growth of BCG after infection of A549 cells. BCG is an attenuated strain of the virulent *M. bovis* and is a live vaccine against tuberculosis. Because BCG bacilli share biological and pathological characteristics [33] and over 99.5% of their genome with that of *M. tuberculosis* [34], BCG is frequently utilized for the analysis of virulence of *M. tuberculosis*.

Utilizing BCG, we first found that exogenously added hyaluronan enhances the growth of BCG after incubation with A549 cells. We found that gentamicin treatment abrogated the growth of both BCG and *M. tuberculosis*, showing that these mycobacteria grow outside A549 cells. By contrast, this BCG strain (Pasteur) and *M. tuberculosis* H37Rv grew inside J774 mouse macrophages. These data apparently suggest that intracellular spaces in A549 cells are not suitable for the growth of mycobacteria.

Mycobacteria are intracellular pathogens and survive in macrophages by blocking phagosome-lysosome fusion (P-L fusion) at the stage of Rab5–Rab7 conversion [35]–[37]. Mycobacteria can infect non-professional epithelial cells in addition to alveolar macrophages. However, the exact mechanisms of how mycobacteria invade and persist or are killed in epithelial cells are unknown. Clemens and Horwitz demonstrated that mycobacterial phagosomes acquired Rab7 in HeLa epithelial cells, suggesting that P-L fusion is not efficiently blocked. Furthermore, Takeda's group recently found that type II pneumocytes produce antimicrobial peptides, secretory leukocyte protease inhibitor and Lipocalin 2, which have potent anti-mycobactericidal activities [5],[6]. Such bactericidal molecules may contribute to the inhibition of intracellular growth of mycobacteria within type II pneumocytes. These data suggest that intracellular trafficking of mycobacteria-containing vacuoles and intracellular states of mycobacteria are different from that in macrophages.

We found that both BCG and *M. tuberculosis* grew in the media containing hyaluronan as the sole carbon source (Figure 2A and 3). In addition to hyaluronan, mammals synthesize several GAGs, but hyaluronan most strongly supported the growth of BCG among GAGs and is comparable with glucose (Figure 2). By contrast, environmental mycobacteria, such as *M. smegmatis* and *M. avium*, failed to use hyaluronan as a carbon source. These data help us to understand why pathogenic mycobacteria have the ability to adhere to hyaluronan and metabolize it. It is reasonable to assume that this property is a great advantage, allowing them to grow in the hyaluronan-rich respiratory organs of their hosts.

Because hyaluronan is a long carbon chain, we considered that cleavage must be an essential step for its use as a carbon source, and indeed found hyaluronidase activity in BCG (Figure 4). Although certain other species of bacterial pathogens, such as *Streptococcus*, *Staphylococcus*, and *Streptomyces*, produce hyaluronidases [38], there has been no report of hyaluronidase of mycobacteria. This is the first report showing hyaluronidase activity in mycobacteria.

There are two main groups of hyaluronidases identified to date. One group is endo-*β*-*N*-acetyl-hexosaminidase or endo-*β*-glucuronidase, which degrades hyaluronan by hydrolysis [39]. These enzymes are distributed in some vertebrates including mouse and human. Others are lyase type hyaluronidase that degrade hyaluronan by *β*-elimination [39]. Bacterial hyaluronidases are lyases, which are unstable but have stronger activity than those of vertebrates, and generate unsaturated products, which is more suitable for energy supply than saturated hyaluronan. Therefore, it is reasonable to consider that mycobacteria have the lyase type of hyaluronidase. Although hyaluronidase is not yet described in the genome of either *M. tuberculosis* [33] or BCG [34], there are approximately 40 lyases. One of these lyases may be responsible for degradation of hyaluronan. Defining which enzyme is responsible for cleavage of hyaluronan is next important issue. Most hyaluronidases in mammals and bacteria display redundancy in recognition of their GAG substrates. Our data show that chondroitin sulfate also supported the growth of BCG (Figure 2). This may imply that hyaluronidase(s) of BCG cleave chondroitin sulfate as well.

Hyaluronan possesses many properties *in vivo* and it is believed that these biological activities are dependent on its size [40]–[42]. Although hyaluronan is composed of simple repeating disaccharides, its secondary structure is flexible. It is affected by the numbers of intramolecular hydrogen bonds, their location, and hydrophobic interactions [43],[44], all of which are increased as the size of the chains increase. Dynamic laser light-scattering analysis showed that the rod-like structure of low molecular weight hyaluronan changes to a stiff coil structure beyond a molecular weight of 1×10^5^ Da [45]. Taken together, it is conceivable that hyaluronan synthesized by HAS1 and HAS3 exhibits a different structure from that synthesized by HAS2. Employing HAS transfectants, we found that both BCG and *M. tuberculosis* utilize hyaluronan synthesized only by HAS1 or HAS3 for multiplication (Figure 5A and 5C).

The fact that BCG and *M. tuberculosis* grow when co-cultured with HAS1 and HAS3 but not HAS2 transfected cells (Figure 5A and 5C) suggests that HAS1 and HAS3-synthesized hyaluronan supports the growth of mycobacteria in the human body. We founds that HAS1 is the major hyaluronan synthase in *M. tuberculosis*-infected mouse lungs (Figure 6A). HAS1 is expressed in immune cells, such as dendritic cells and T cells [46]. To clarify what kind of cell expresses HAS1 during mycobacterial infection is the next important issue.

In spite of the importance of hyaluronan in host protection in the lungs, its role in mycobacterial diseases had not been elucidated. In this study, we demonstrated that BCG and *M. tuberculosis* can utilize it as a carbon source. Hyaluronan was observed in the granulomatous region of mice lungs infected with *M. tuberculosis* (Figure 6). Furthermore, *M. tuberculosis* bacilli were residing in the region where hyaluronan was located in the lungs of monkeys that had died from tuberculosis (Figure 7). We also showed that blocking hyaluronidase inhibited *in vivo* multiplication of BCG (Figure 8). These results suggest that pathogenic mycobacteria have evolved to exploit the intrinsically host-protective molecule, hyaluronan as a nutrient to grow. Similar behavior of pathogenic mycobacteria was observed during infection of macrophages, that is, BCG is phagocytized in a cholesterol-dependent manner [47] and utilizes cholesterol as a carbon source to survive in activated macrophages [48]. It is likely that mycobacteria developed several strategies to obtain nutrients under nutrient-limited conditions.

After digestion of hyaluronan, it must be incorporated into mycobacteria through specific receptors or membrane proteins. Based on our results and consideration, hyaluronidase and a potential transporter of fragmented hyaluronan of pathogenic mycobacteria are potential drug targets.

## Materials and Methods

### Animal studies

All animals were maintained under specific pathogen-free conditions in the animal facilities of Osaka City University Graduate School of Medicine and in a biosafety-level-3 facility at The Research Institute of Tuberculosis according to the standard guidelines for animal experiments at each institute.

### Culture medium and reagents

RPMI 1640 media, L-glutamine, fetal bovine serum, HEPES, hyaluronan from human umbilical cord, heparin from porcine intestinal mucosa and heparan sulfate from bovine kidney were purchased from Sigma-Aldrich (St. Louis, MO). Chondroitin sulfate A and C were purchased from Calbiochem (Gibbstown, NJ). For conventional culture of mycobacteria, Middlebrook 7H9 medium (Becton Dickinson) supplemented with 0.085% NaCl, 10% albumin-dextrose-catalase (BD Biosciences), 0.2% glycerol, and 0.05% Tween 80 (7H9-ADC) or 7H11-agar supplemented with 0.085% NaCl, 10% oleic acid-albumin-dextrose-catalase (BD Biosciences), and 0.2% glycerol (7H11-OADC) were used. 7H9 medium (Becton Dickinson) supplemented with 0.085% NaCl and 0.1% albumin was used as a carbon-starved 7H9 medium.

### Effect of hyaluronan on extracellular growth of BCG and *M. tuberculosis* after infection to A549 cells

A549 cells were grown in RPMI 1640 medium containing 10% heat-inactivated fetal bovine serum, 2 mM L-glutamine, 25 mM HEPES and 5.5×10^−5^ M 2-mercaptoethanol (complete culture medium) at 37°C in an atmosphere of 5% CO_2_. Cells were suspended at 2×10^5^/ml in complete culture medium and 1 ml of cell suspension was dispensed into individual wells of a 24-well polystyrene plate (BD Biosciences, San Jose, CA). Plates were incubated at 37°C for 24 h and were washed with serum-free RPMI 1640 medium to remove nonadherent cells. Wells were then refilled with 1 ml of complete culture medium. *M. bovis* BCG or *M. tuberculosis* cell suspension was prepared as described previously [1]. The bacterial cell suspension was added to A549 cells at multiplicities of infection (MOI) of 10. After 16 (BCG) or 4 (*M. tuberculosis*) h incubation, unbound bacteria were removed by washing with serum-free RPMI 1640 three times. After adding 1 ml of fresh complete culture medium to each well, hyaluronan solution was added to final concentrations ranging from 5 to 500 µg/ml. Cells were collected periodically for luciferase or CFU assays.

### Luciferase assays

Construction of BCG expressing luciferase was described previously [1]. Luciferase activity was measured using the luciferase assay system from Promega (Madison, WI) according to the manufacturer's protocol on a Wallac 1420 manager as described previously [14].

### Effect of gentamicin on mycobacterial growth after infection to A549 cells

A549 cells in 96-well polystyrene plates (8×10^4^/well) were infected with BCG-Luc or *M. tuberculosis* at MOI of 10 at 37°C. After 16 (BCG) or 4 (*M. tuberculosis*) h, the monolayers were washed three times with RPMI 1640 medium to remove extracellular bacteria. Fresh complete culture medium containing 1 mg/ml of hyaluronan and 50 µg/ml of gentamicin were added to each well (200 µl/well) and incubated at 37°C. Cells were collected periodically for detection of luciferase activity of BCG-Luc or CFU assay of *M. tuberculosis*.

### Evaluation of glucose and GAG as carbon sources for growth of mycobacteria

BCG-Luc or *M. tuberculosis* was adjusted to a concentration of 1×10^4^ CFU/ml in carbon-starved 7H9 medium described previously [14], and 200 µl of bacterial cell suspension was added to 96-well polystyrene plates. Heparin, heparan sulfate, chondroitin sulfate, hyaluronan or glucose was added to appropriate wells to a final concentration of 500 µg/ml. Plates were incubated at 37°C and bacterial cells were collected periodically for detection of luciferase activity of BCG-Luc or CFU assay of *M. tuberculosis*.

### Evaluation of ingestion of hyaluronan into mycobacteria

BCG Pasteur was grown aerobically in 7H9-ADC medium at 37°C. Cells were then collected by centrifugation and half of the cells were heat-killed by heating at 65°C for 30 min. Then bacteria were washed, resuspended by carbon-starved 7H9 medium and adjusted to an optical density at 600 nm of 0.07. One hundred microliters of cell suspension was added to 100 ml of carbon-starved 7H9 with or without 6 mg of ^3^H-labeled hyaluronan and 14 mg of non-labeled hyaluronan (final concentration of 100 mg/L of total hyaluronan). Cells were then incubated at 37°C. After incubation, cells were harvested by use of a Scatron Harvester (Scatron) onto a glass fiber filter. The incorporated radioactivity was measured in a gamma counter (ALOKA ARC-2000).

### Effect of hyaluronan on mycobacterial growth

*M. tuberculosis* strain H37Rv, *M. smegmatis* strain mc^2^155 and *M. avium* strain type4 were grown in carbon-starved 7H9 medium containing 0.5 mg/ml of hyaluronan, and the cultures were monitored periodically for their optical density at 600 nm (*M. tuberculosis and M. smegmatis*) or CFU (*M. tuberculosis* and *M. avium*).

### Preparation of oligosaccharides from hyaluronan digested by crude extracts of BCG

BCG was grown in 7H9-ADC medium to mid-log phase. After incubation, bacterial cells were harvested, washed three times with ice-cold PBS (pH 6.0) and resuspended in the same buffer. To disrupt bacterial cells, the cell suspension was added to a screw-capped tube containing glass beads (diameter, 1.0 mm) and the tube was oscillated on a Mini-Bead Beater (Cole-Parmer). The tube was centrifuged at 10,000×*g* for 10 min, and the supernatant containing the bacterial protein extract was collected into a new tube. The protein solution was then mixed with 1 mg/ml of hyaluronan in PBS (pH 6.0) at 37°C. After incubation for 24 h, the solution was mixed with an equal volume of phenol to remove protein. The mixture was centrifuged at 10,000×*g* for 10 min and the supernatant was collected for PAGE analysis.

### Polyacrylamide Gel Electrophoresis (PAGE) of hyaluronan

PAGE analysis of hyaluronan was performed as previously described by Ikegami-Kawai *et al.* [30] with minor modifications. The PAGE mini-slab gels contained 12.5% acrylamide, 0.32% *N, N′*-methylene bis-acrylamide in 0.1 M Tris-borate-1 mM Na_2_EDTA (TBE, pH 8.3). For the electrophoretic run, samples containing hyaluronan were mixed with one-fifth volume of 2M sucrose in TBE and 10 µl of the mixtures was applied directly to the gel. Bromophenol blue in TBE containing 0.3 M sucrose was used as a tracking dye, but was generally applied to a well with no sample. The gels were electrophoresed at 300 V for approximately 70 min using TBE as a reservoir buffer. After electrophoresis, the gels were stained with alcian blue as described previously [30]. Briefly, the gels were soaked in 0.05% Alcian blue in distilled water for 30 min in the dark and destained in water for 30 min.

### Inhibition of bacterial growth by hyaluronidase inhibitor

BCG-Luc or *M. tuberculosis* H37Rv was suspended in 7H9-ADC, carbon-starved 7H9 or carbon-starved 7H9 containing 0.5 mg/ml of hyalurona to a final concentration of 1×10^4^ CFU/ml and 200µl of each suspension was added to 96-well polystyrene plates. Vcpal was added to each well. Bacterial cells were then incubated at 37°C and were collected periodically for detection of luciferase activity for BCG-Luc or CFU assay for *M. tuberculosis*. Similarly, *M. tuberculosis* H37Rv was incubated in the media containing 0.5 mg/ml hyaluronan in presence or absence of 0.1 or 0.5 mM of apigenin or quercetin. After incubation for 7 days, living bacterial number was determined by CFU assay.

### RT-PCR

The expression of hyaluronan synthase genes in the lung tissues of mice aerogenically challenged with the virulent *M. tuberculosis* strain H37Rv was determined by RT-PCR. Seven-week-old of female BALB/c mice were aerogenically infected with the *M. tuberculosis* strain H37Rv (2×10^2^ CFU/mouse) using a Glas-Col chamber. At different time points, 3 mice per group were euthanized and, the lungs were homogenized in PBS containing 0.05% Tween 80. The homogenates were centrifuged, and the pellets were processed to isolate total RNA using the RNeasy mini kit (QIAGEN, West Sussex, UK) according to the manufacturer's instruction. One microgram of total RNA was reverse transcribed using Super Script II RNase H reverse transcriptase (Invitrogen). The cDNA was then subjected to RT-PCR. The following primer pairs were used: β-actin, 5′-TGGAATCCTGTGGCATCCATGAAAC-3′ (F) and 5′-TAAACGCAGCAGCTCAGTAACAGTCCG-3′ (R); HAS1, 5′-GCTCTATGGGGCGTTCCTC-3′ (F) and 5′-CACACATAAGTGGCAGGGTCC-3′ (R); HAS2, 5′-TGGAACACCGGAAAATGAAGAAG-3′ (F) and 5′-GGACCGAGCCGTGTATTTAGTTGC-3′ (R); HAS3, 5′-CCATGAGGCGGGTGAAGGAGAG-3′ (F) and 5′-ATGCGGCCACGGTAGAAAAGTTGT-3′ (R). The amplification procedure involved initial denaturation at 94°C for 4 min followed by 35 cycles of denaturation at 94°C for 1 min, annealing of primers at 57°C for 1 min and primer extension at 72°C for 3 min. After completion of the 35th cycle, the extension reaction was continued for another 7 min at 72°C.

Total RNA was extracted from A549 cells by RNeasy mini kit (QIAGEN) and then 1 µg of total RNA was reverse transcribed using Super Script II RNase H reverse transcriptase (Invitrogen). The cDNA was then subjected to RT-PCR. The following primer pairs were used: β-actin, 5′-GATCATTGCTCCTCCTGAGC-3′ (F) and 5′-CACCTTCACCGTTCCAGTTT-3′ (R); HAS1, 5′- ACTCGGACACAAGGTTGGAC -3′ (F) and 5′- TGTACAGCCACTCACGGAAG -3′ (R); HAS2, 5′- ATGCATTGTGAGAGGTTTCT -3′ (F) and 5′- CCATGACAACTTTAATCCCAG -3′ (R); HAS3, 5′- GACGACAGCCCTGCGTGT -3′ (F) and 5′- TTGAGGTCAGGGAAGGAGAT-3′ (R). The amplification procedure involved initial denaturation at 94°C for 10 min followed by 40 cycles of denaturation at 94°C for 1 min, annealing of primers at 56°C for 1 min and primer extension at 72°C for 2.5 min.

### Lung sections of rhesus monkeys that died from tuberculosis

The *M. tuberculosis* H37Rv challenge infection study of in rhesus male monkeys was performed previously [49]. The lung of non-vaccinated monkeys that died of tuberculosis 3 month after intratracheal challenge of 3,000 CFU/lung of *M. tuberculosis* H37Rv were immediately removed and fixed with 15% formalin for 10 days. Three animals' lungs were embedded in paraffin blocks and used in this study as well.

### Histochemical staining for hyaluronan

After deparaffinization by washing with xylene and ethanol, the tissue sections were washed in TBS and incubated with fresh TBE containing 0.05 mM of Pronase K (Dako) for 60 min at room temperature. After washing with TBS containing 1% bovine serum albumin, the slides were incubated with 3% bovine serum albumin in TBS for 30 min at room temperature to block non-specific binding sites. The slides were then washed with TBS twice for 10 min and incubated with the biotinylated hyaluronan-binding protein (HABP) probe at a concentration of 2 mg/ml in TBS for 60 min at room temperature. Following washing in TBS, the slides were incubated with a streptavidin-peroxidase reagent and the staining developed using DAKO Cytomation LSAB-system AP (Dako). The slides were then washed with distilled water and counterstained with Mayer's hematoxylin. Paraffin sections were also stained with alcian blue (Sigma) pH 2.5 (3% acetic acid) for 5 min. The slides were counterstained with nuclear fast red (Biomeda) and mounted with Gel/Mount (Biomeda). For GAG digestion, 0.5 mg/ml (10 U/ml) *Streptomyces* hyaluronidase was added for 30 min at 37°C before alcian blue staining. The slides were stained by Ziehl-Neelsen technique using carbol-fuchsin and malachite green (Sigma).

## Supporting Information

Figure S1 Apigenin and quercetin suppress growth of *M. tuberculosis* in the media containing hyaluronan as a sole carbon source. *M. tuberculosis* H37Rv was cultured for 7 days in carbon-starved media (7H9) or the media containing 500 µg/ml hyaluronan as a sole carbon source (7H9-HA). Apigenin or quercetin, inhibitors of hyaluronidase, were added to be 0.5 mM or 0.1 mM. CFU was determined at time 0 (open bar) and 7 days after culture (closed bars).
(0.08 MB TIF)

Figure S2 Analysis of transcription of HAS genes in A549 cells. Total RNA was extracted from A549 cells cultured in RPMI1640 media containing 10% FCS. Transcription of each gene encoding human HAS1, HAS2, HAS3 and beta-actin was analyzed by RT-PCR. Three samples were analyzed and representative data are presented. M, DNA markers.
(0.61 MB TIF)

## References

[1] Aoki K, Matsumoto S, Hirayama Y, Wada T, Ozeki Y (2004). Extracellular mycobacterial DNA-binding protein 1 participates in *Mycobacterium*-lung epithelial cell interaction through hyaluronic acid.. J Biol Chem.

[2] Bermudez LE, Goodman J (1996). *Mycobacterium tuberculosis* invades and replicates within type II alveolar cells.. Infect Immun.

[3] Hernandez-Pando R, Jeyanathan M, Mengistu G, Aguilar D, Orozco H (2000). Persistence of DNA from *Mycobacterium tuberculosis* in superficially normal lung tissue during latent infection.. Lancet.

[4] Teitelbaum R, Schubert W, Gunther L, Kress Y, Macaluso F (1999). The M cell as a portal of entry to the lung for the bacterial pathogen *Mycobacterium tuberculosis*.. Immunity.

[5] Nishimura J, Saiga H, Sato S, Okuyama M, Kayama H (2008). Potent antimycobacterial activity of mouse secretory leukocyte protease inhibitor.. J Immunol.

[6] Saiga H, Nishimura J, Kuwata H, Okuyama M, Matsumoto S (2008). Lipocalin 2-dependent inhibition of mycobacterial growth in alveolar epithelium.. J Immunol.

[7] Dannenberg AM (1994). Roles of cytotoxic delayed-type hypersensitivity and macrophage-activating cell-mediated immunity in the pathogenesis of tuberculosis.. Immunobiology.

[8] Gobin J, Horwitz MA (1996). Exochelins of *Mycobacterium tuberculosis* remove iron from human iron-binding proteins and donate iron to mycobactins in the *M. tuberculosis* cell wall.. J Exp Med.

[9] Ernst JD (1998). Macrophage receptors for *Mycobacterium tuberculosis*.. Infect Immun.

[10] Menozzi FD, Rouse JH, Alavi M, Laude-Sharp M, Muller J (1996). Identification of a heparin-binding hemagglutinin present in mycobacteria.. J Exp Med.

[11] Pethe K, Alonso S, Biet F, Delogu G, Brennan MJ (2001). The heparin-binding haemagglutinin of *M. tuberculosis* is required for extrapulmonary dissemination.. Nature.

[12] Soares de Lima C, Zulianello L, Marques MA, Kim H, Portugal MI (2005). Mapping the laminin-binding and adhesive domain of the cell surface-associated Hlp/LBP protein from *Mycobacterium leprae*.. Microbes Infect.

[13] Pethe K, Aumercier M, Fort E, Gatot C, Locht C (2000). Characterization of the heparin-binding site of the mycobacterial heparin-binding hemagglutinin adhesin.. J Biol Chem.

[14] Katsube T, Matsumoto S, Takatsuka M, Okuyama M, Ozeki Y (2007). Control of cell wall assembly by a histone-like protein in mycobacteria.. J Bacteriol.

[15] Itano N, Sawai T, Yoshida M, Lenas P, Yamada Y (1999). Three isoforms of mammalian hyaluronan synthases have distinct enzymatic properties.. J Biol Chem.

[16] Shyjan AM, Heldin P, Butcher EC, Yoshino T, Briskin MJ (1996). Functional cloning of the cDNA for a human hyaluronan synthase.. J Biol Chem.

[17] Weigel PH, DeAngelis PL (2007). Hyaluronan synthases: a decade-plus of novel glycosyltransferases.. J Biol Chem.

[18] Weigel PH, Hascall VC, Tammi M (1997). Hyaluronan synthases.. J Biol Chem.

[19] Camenisch TD, Spicer AP, Brehm-Gibson T, Biesterfeldt J, Augustine ML (2000). Disruption of hyaluronan synthase-2 abrogates normal cardiac morphogenesis and hyaluronan-mediated transformation of epithelium to mesenchyme.. J Clin Invest.

[20] Aruffo A, Stamenkovic I, Melnick M, Underhill CB, Seed B (1990). CD44 is the principal cell surface receptor for hyaluronate.. Cell.

[21] Yang B, Hall CL, Yang BL, Savani RC, Turley EA (1994). Identification of a novel heparin binding domain in RHAMM and evidence that it modifies HA mediated locomotion of ras-transformed cells.. J Cell Biochem.

[22] Bartolazzi A, Peach R, Aruffo A, Stamenkovic I (1994). Interaction between CD44 and hyaluronate is directly implicated in the regulation of tumor development.. J Exp Med.

[23] Hall CL, Yang B, Yang X, Zhang S, Turley M (1995). Overexpression of the hyaluronan receptor RHAMM is transforming and is also required for H-ras transformation.. Cell.

[24] Jameson JM, Cauvi G, Sharp LL, Witherden DA, Havran WL (2005). Gammadelta T cell-induced hyaluronan production by epithelial cells regulates inflammation.. J Exp Med.

[25] Jiang D, Liang J, Fan J, Yu S, Chen S (2005). Regulation of lung injury and repair by Toll-like receptors and hyaluronan.. Nat Med.

[26] Teder P, Vandivier RW, Jiang D, Liang J, Cohn L (2002). Resolution of lung inflammation by CD44.. Science.

[27] Termeer C, Benedix F, Sleeman J, Fieber C, Voith U (2002). Oligosaccharides of hyaluronan activate dendritic cells via toll-like receptor 4.. J Exp Med.

[28] Forteza R, Lieb T, Aoki T, Savani RC, Conner GE (2001). Hyaluronan serves a novel role in airway mucosal host defense.. FASEB J.

[29] Jacobs WR, Barletta RG, Udani R, Chan J, Kalkut G (1993). Rapid assessment of drug susceptibilities of *Mycobacterium tuberculosis* by means of luciferase reporter phages.. Science.

[30] Ikegami-Kawai M, Takahashi T (2002). Microanalysis of hyaluronan oligosaccharides by polyacrylamide gel electrophoresis and its application to assay of hyaluronidase activity.. Anal Biochem.

[31] Botzki A, Rigden DJ, Braun S, Nukui M, Salmen S (2004). L-Ascorbic acid 6-hexadecanoate, a potent hyaluronidase inhibitor. X-ray structure and molecular modeling of enzyme-inhibitor complexes.. J Biol Chem.

[32] Li MW, Yudin AI, VandeVoort CA, Sabeur K, Primakoff P (1997). Inhibition of monkey sperm hyaluronidase activity and heterologous cumulus penetration by flavonoids.. Biol Reprod.

[33] Cole ST, Brosch R, Parkhill J, Garnier T, Churcher C (1998). Deciphering the biology of *Mycobacterium tuberculosis* from the complete genome sequence.. Nature.

[34] Brosch R, Gordon SV, Garnier T, Eiglmeier K, Frigui W (2007). Genome plasticity of BCG and impact on vaccine efficacy.. Proc Natl Acad Sci U S A.

[35] Rink J, Ghigo E, Kalaidzidis Y, Zerial M (2005). Rab conversion as a mechanism of progression from early to late endosomes.. Cell.

[36] Deretic V, Singh S, Master S, Harris J, Roberts E (2006). *Mycobacterium tuberculosis* inhibition of phagolysosome biogenesis and autophagy as a host defence mechanism.. Cell Microbiol.

[37] Via LE, Deretic D, Ulmer RJ, Hibler NS, Huber LA (1997). Arrest of mycobacterial phagosome maturation is caused by a block in vesicle fusion between stages controlled by rab5 and rab7.. J Biol Chem.

[38] Girish KS, Kemparaju K (2007). The magic glue hyaluronan and its eraser hyaluronidase: a biological overview.. Life Sci.

[39] Stern R, Jedrzejas MJ (2006). Hyaluronidases: their genomics, structures, and mechanisms of action.. Chem Rev.

[40] Hascall VC, Majors AK, De La Motte CA, Evanko SP, Wang A (2004). Intracellular hyaluronan: a new frontier for inflammation?. Biochim Biophys Acta.

[41] Jiang D, Liang J, Noble PW (2007). Hyaluronan in tissue injury and repair.. Annu Rev Cell Dev Biol.

[42] Stern R, Kogan G, Jedrzejas MJ, Soltes L (2007). The many ways to cleave hyaluronan.. Biotechnol Adv.

[43] Gribbon P, Heng BC, Hardingham TE (2000). The analysis of intermolecular interactions in concentrated hyaluronan solutions suggest no evidence for chain-chain association.. Biochem J.

[44] Scott JE, Heatley F (1999). Hyaluronan forms specific stable tertiary structures in aqueous solution: a 13C NMR study.. Proc Natl Acad Sci USA.

[45] Almond A, Brass A, Sheehan JK (1998). Deducing polymeric structure from aqueous molecular dynamics simulations of oligosaccharides: predictions from simulations of hyaluronan tetrasaccharides compared with hydrodynamic and X-ray fibre diffraction data.. J Mol Biol.

[46] Mummert ME, Mummert D, Edelbaum D, Hui F, Matsue H (2002). Synthesis and surface expression of hyaluronan by dendritic cells and its potential role in antigen presentation.. J Immunol.

[47] Gatfield J, Pieters J (2000). Essential role for cholesterol in entry of mycobacteria into macrophages.. Science.

[48] Pandey AK, Sassetti CM (2008). Mycobacterial persistence requires the utilization of host cholesterol.. Proc Natl Acad Sci U S A.

[49] Sugawara I, Sun L, Mizuno S, Taniyama T (2009). Protective efficacy of recombinant BCG Tokyo (Ag85A) in rhesus monkeys (Macaca mulatta) infected intratracheally with H37Rv *Mycobacterium tuberculosis*.. Tuberculosis.

