# Supplementary figures and images for: Mycobacteria Exploit Host Hyaluronan for Efficient Extracellular Replication
Source: PLoS Pathog. 2009 Oct 30;5(10):e1000643. doi: 10.1371/journal.ppat.1000643 (PMC2763203; doi:10.1371/journal.ppat.1000643)

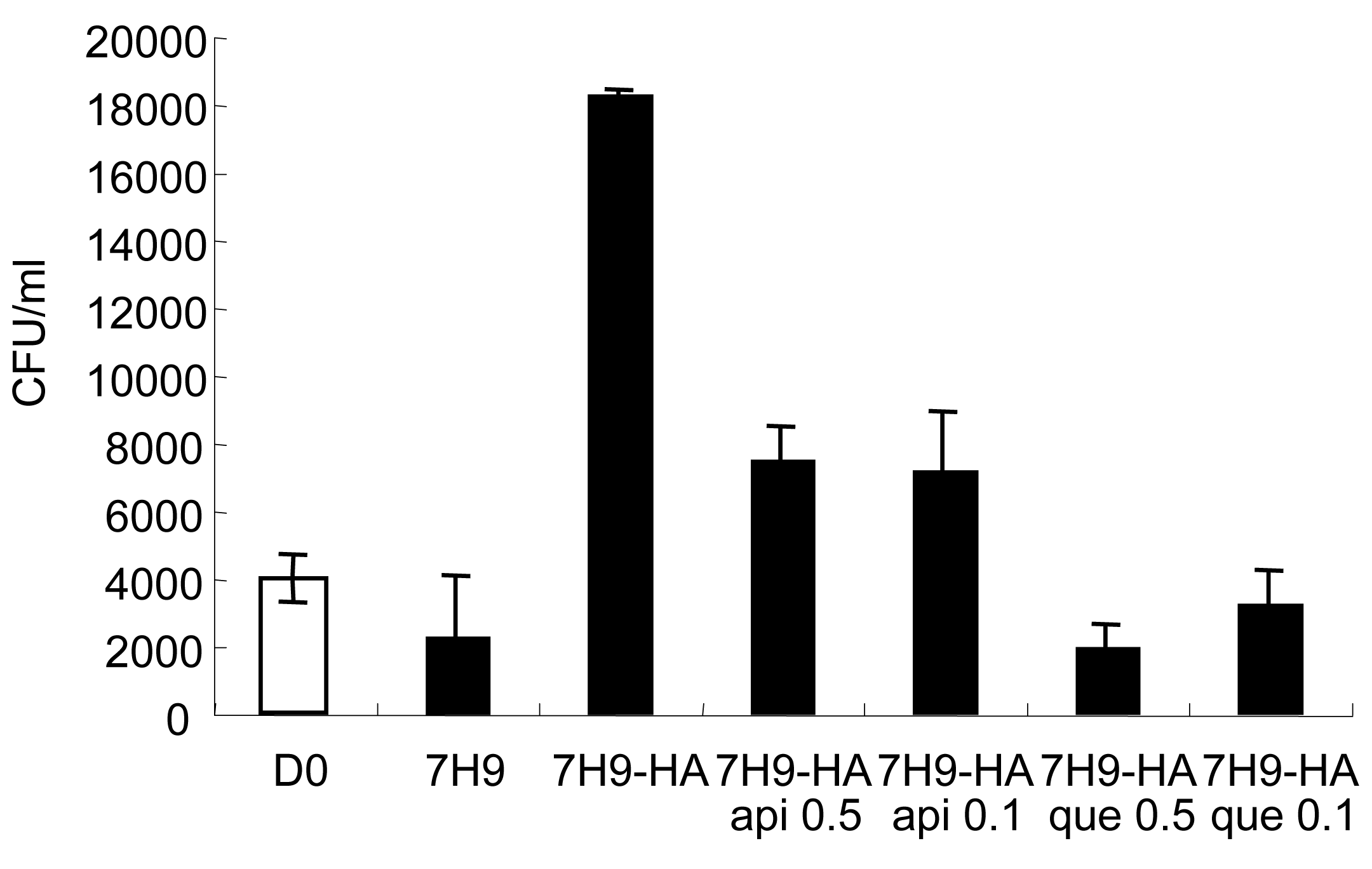

Supplement: Figure S1 — Apigenin and quercetin suppress growth of M. tuberculosis in the media containing hyaluronan as a sole carbon source. M. tuberculosis H37Rv was cultured for 7 days in carbon-starved media (7H9) or the media containing 500 µg/ml hyaluronan as a sole carbon source (7H9-HA). Apigenin or quercetin, inhibitors of hyaluronidase, were added to be 0.5 mM or 0.1 mM. CFU was determined at time 0 (open bar) and 7 days after culture (closed bars). (0.08 MB TIF) [file ppat.1000643.s001.tif]

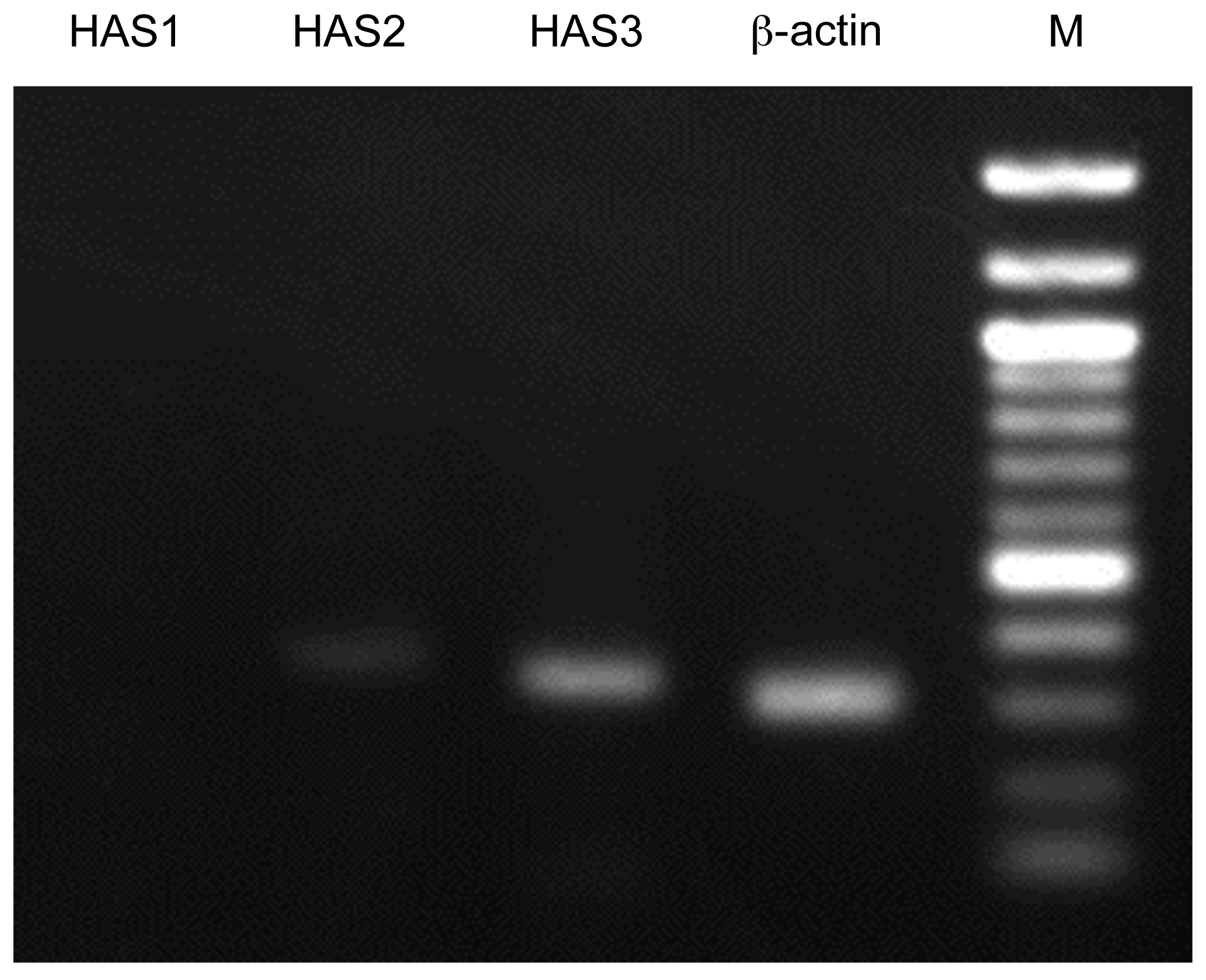

Supplement: Figure S2 — Analysis of transcription of HAS genes in A549 cells. Total RNA was extracted from A549 cells cultured in RPMI1640 media containing 10% FCS. Transcription of each gene encoding human HAS1, HAS2, HAS3 and beta-actin was analyzed by RT-PCR. Three samples were analyzed and representative data are presented. M, DNA markers. (0.61 MB TIF) [file ppat.1000643.s002.tif]
